# Biodegradation of Carbon Tetrachloride in Groundwater: Microbial Community Shifts and Functional Genes Involvement in Enhanced Reductive Dechlorination

**DOI:** 10.3390/toxics13080704

**Published:** 2025-08-21

**Authors:** Zhengwei Liu, Mingbo Sun, Wei Wang, Shaolei Zhao, Yan Xie, Xiaoyu Lin, Jingru Liu, Shucai Zhang

**Affiliations:** 1 State Key Laboratory of Chemical Safety, Qingdao 266061, China; liuzw.qday@sinopec.com (Z.L.); sunmb.qday@sinopec.com (M.S.); wangw.qday@sinopec.com (W.W.); zhaosl.qday@sinopec.com (S.Z.); xiey.qday@sinopec.com (Y.X.); linxy.qday@sinopec.com (X.L.); liujr.qday@sinopec.com (J.L.); 2 SINOPEC Research Institute of Safety Engineering Co., Ltd., Qingdao 266061, China

**Keywords:** carbon tetrachloride, bioremediation, reductive dechlorination, microbial community, groundwater contamination

## Abstract

Carbon tetrachloride (CT) is a toxic volatile chlorinated hydrocarbon, posing a serious hazard to ecosystem and human health. This study discussed the bioremediation possibility of groundwater contaminated by CT. Enhanced reductive dechlorination bioremediation (ERD) was used to promote the reductive dechlorination process of CT by adding yeast extract as a supplementary electron donor. The microcosm samples of the Control and Experi group were setup in the experiment, and the CT degradation efficiency and microbial community structure changes over 150 days were monitored. The results showed that the Experi group achieved complete degradation of CT within 40 days, while the control group had no significant change. By analyzing the physical and chemical indexes such as VFAs, sulfate ions, oxidation–reduction potential, pH value and so on, the key changes in the degradation process of CT were revealed. Microbial community analysis showed that specific microorganisms such as *Acinetobacter johnsonii*, *Aeromonas media* and *Enterobacter mori* played a significant role in the degradation of CT. They may produce hydrogen through fermentation to provide electron donors for the reductive dechlorination of CT. In addition, the genes of reductive dehalogenase synthase related to CT degradation were also identified, which provided molecular evidence for understanding the biodegradation mechanism of CT. The results deliver a scientific basis for optimizing the bioremediation strategy of CT-contaminated groundwater.

## 1. Introduction

Carbon tetrachloride (CT) is a toxic volatile chlorinated hydrocarbon with a special odor and strong volatility. It is used as an excellent solvent in laboratories and is often used as a raw material for paints and rubber or as a degreasing agent for fire extinguishers in industry [1]. CT has a significant impact on ecosystems and is a typical carcinogenic, teratogenic and mutagenic substance [2,3,4]. Thus, CT is identified as a common co-contaminant in ~20% of the Superfund sites in America and has been characterized as one of the top priority hazardous contaminants [5]. According to the “Hygienic Standards for Drinking Water” (GB 5749-2022), the maximum limit of CT in drinking water is 2 μg/L [6]. CT contamination has been found in groundwater and soil in multiple regions of Spain, China, USA, etc. [3,4,5].

However, satisfactory CT remediation is challenging to achieve upon most natural attenuations and engineering implementations. Several investigations have revealed the efficacy of in situ chemical reduction techniques [7,8], chemical oxidation [9,10,11] and bioremediation technology [3,4,12,13,14,15,16] for the remediation of groundwater contaminated with CT. Among these above, in situ enhanced reductive dichlorination (ERD) presents the advantage of being cost-efficient and environmentally friendly.

Stari and his coworkers stated the enrichment of a steady aerobic CT-degrading microbial consortium from CT-contaminated groundwater, able to degrade 30 μM of CT in a week [12]. Chloroform (CF) or other low-chlorinated compounds were not reorganized during the CT metabolism. Koenig and his coworkers described the sequential utilization of SO_4_^2−^ and dehalo-respiration as a microbial approach for the conversion of CT (10 μM) and PCE (14 μM). S^2^ generation in a *Desulfovibrio vulgaris* culture resulted in complete CT disappearance in 12 days [15]. Liu and his coworkers formulated a three-layer PRB material containing Fe^0^ and coconut-shell biochar, effectively applied at a CF-, CT-, DCM- and 1,2-dichloroethane-contaminated site. The devised PRB consistently discharged organic carbon and Fe, promoting the CH dechlorinating bacteria propagation. The removal efficiencies were 95.7–99.6% for CT throughout the 250-day PRB setup period [4].

However, the possible degradation process and the microbial community changes at different reactive stages throughout the CT biodegradation under strongly reducing conditions are ambiguous. To address this disparity, CT-contaminated groundwater from a petrochemical plant was collected to constitute microcosm experiments. The electron donor yeast extract powder was supplemented to explore the viability and possible degradation mechanisms of enhanced reductive dechlorination for remediating actual CT-contaminated groundwater. This study also means to assess the shift in the microbial community structure and recognize the crucial bacteria that are most effective in the CT biodegradation process.

## 2. Materials and Methods

### 2.1. Sampling

**Figure 1:**
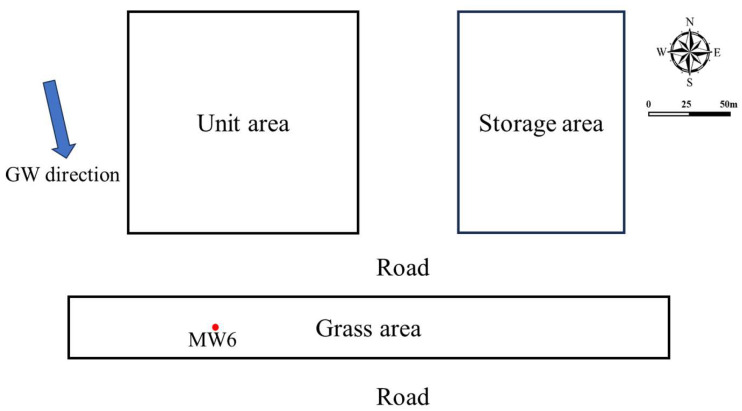
The layout of groundwater sampling points.

Groundwater contaminated by CT was gathered. The investigation site is located in a petrochemical plant in central China that has been operational for over 40 years. Figure 1 displays the layout of the contaminated site as well as the monitoring well positioning. The aquifer medium was mainly composed of silt in the depth range. Primarily, the groundwater type is Quaternary phreatic groundwater from 2 m to 13 m beneath the surface. The natural groundwater flow direction was principally from the north towards the south. Groundwater (MW6) was gathered using Micro-Purge groundwater sampling equipment (Sample PRO, QED Environmental Systems Limited Inc., 2355 Bishop Circle West Dexter, MI 48130, USA) after the well washing.

### 2.2. Microcosm Sample Setup

Two microcosm samples, Control and Experi, were established, and their constituents are shown in Table 1. Groundwater containing chlorinated hydrocarbons and benzene contaminants was completely removed by nitrogen blowing, while preserving the original microbial community structure as much as possible. The anaerobic microcosm was assembled in a 50 mL glass serum bottle; each was stuffed to capacity with the gathered groundwater (refill contaminant CT) and YE. The contaminant was added to the deaired groundwater to attain the targeted concentrations. In the Control microcosm sample, an additional 10 mg/L HgCl_2_ was supplemented to inhibit microbial activity [17]. The microcosm was prepared in an N_2_ glovebox. The microcosm sample experiment was performed over a 150-day period.

### 2.3. Analytical Methods

Dissolved oxygen (DO), pH and oxidation–reduction potential (ORP) were quantified by in situ portable equipment. Ion Chromatography (DIONEX ICS-900, Thermo Fisher Scientific, Lakeside Drive, Sunnyvale, CA, USA) was employed to determine SO_4_^2−^ and NO_3_^−^. Cations including Fe^2+^ and Mn^2+^ were measured using a portable HACH DR3900 analyzer following the standard methods 8146 (1,10-phenanthroline photometric method) and 8034 (periodate method), respectively. The volatile organic compound concentrations were analyzed employing purge-trap and gas chromatography–mass spectrometry (GC-MS 7890B-5977B, Agilent, Santa Clara City, CA, USA), following the US EPA Method 502.2 procedures [18]. All the analysis tests above were conducted in triplicate.

### 2.4. Microbial Community

The microbe was filtrated via the 0.22 μm filter membranes and deposited at −85 °C. For 16 S rRNA gene sequencing, the V4 region of the 16 S rRNA gene was amplified using primers 515 F (5′-GTGCCAGCMGCCGCGGTAA-3′) and 806 R (5′-GGACTACHVGGGTWTCTAAT-3′). Sequencing was carried out on HiSeq 4000 platform (Illumina Inc., San Diego, CA, USA). Raw sequences were filtered, and high-quality reads were assigned to OTUs, and then annotated via RDP classifier compared to the SILVA 128/16S database. The phylogenetic tree was created to reveal the phylogenetic position and the known CT reductive dehalogenation bacteria, using the FastTree (version 2.1.3) in genus level.

Entire DNA was isolated using a Fast DNA Stool Mini Kit (Qiagen, Hilden, Germany). DNA integrity and concentration were weighed by Qubit (Thermo Fisher Scientific, Waltham, MA, USA), NanoDrop2000 spectrophotometer (Thermo Fisher Scientific, Waltham, MA, USA) and agarose gel electrophoresis, respectively. The libraries were constructed using VAHTS Universal PLUS DNA Library Prep Kit (Nanjing Vazyme Biotech Co., Ltd., Nanjing, China). The analysis was managed by OE Biotech Co., Ltd. (Shanghai, China).

The libraries were sequenced on a lllumina NovaSeqX Plus platform/DNBSEQ-T7 and 150 bp paired-end reads were created. Sequences in the FastQ file were filtered using fastp (v 0.20.1). The post-filtered pair-end reads were aligned using bbmap (v 38.93-0). Metagenome assembly was implemented using MEGAHIT (v 1.2.9) after acquiring valid reads. We used gaps inside the scaffold as breakpoints to interrupt the scaffold into new contigs (Scaftig), and these new Scaftigs with length > 500 bp were reserved. ORF prediction of the assembled scaffolds using prodigal (v 2.6.3) was performed and translated into amino acid sequences. The non-redundant gene sets were created for all predicted genes using MMSeqs2 (v 13.4511). The longest gene was selected as a representative sequence of each gene set. Clean reads were aligned against the non-redundant gene set (95% identity) using salmon (v 1.8.0), and the gene abundant information in the corresponding sample was counted. The species taxonomy was obtained as a consequence of the NR Library taxonomy database, and the species abundance was computed using the gene’s abundance. In order to construct the corresponding taxonomy abundance profile, abundance statistics were performed at each level of Domain, Kingdom, Phylum, Class, Order, Family, Genus and Species. A representative nucleotide sequence was selected for each gene set within the non-redundant gene catalog. These representative nucleotide sequences were then translated into amino acid sequences. Functional annotation of the resulting amino acid sequences was performed against the NR, KEGG, eggNOG, SWISS-PROT and GO databases using DIAMOND (v2.1.3) with an e-value cutoff of 1 × 10^−5^.

The gene sets were compared to the CAZy database using hmmscan (v 3.1) to attain the carbohydrate active enzyme information corresponding to the gene, and then the carbohydrate activity was calculated using the sum of the gene abundances corresponding to the carbohydrate active enzyme abundance.

The taxonomy abundance spectrum PCoA plotting was carried out using R software (v 4.1.2), and the results of the equidistant matrix of PCoA and NMDS were analyzed. Then the R package (v 4.1.2) was utilized to investigate the differences between different groups using ANOVA/Kruskal–Wallis/*t* test/Wilcoxon statistical tests. The linear discriminant analysis effect size (LEfSe) method was used to compare the taxonomy abundance spectrum.

## 3. Results and Discussion

### 3.1. Physicochemical Properties of the Groundwater

The physicochemical properties of the collected groundwater in the microcosm sample are shown in Table 2. The groundwater was neutral in a reducing condition, as verified by pH 6.87 and ORP −38 mV. The DO and NO_3_^−^ concentrations, acting as electron acceptors in microbial-mediated redox reactions, were low. The major contaminants were CT, 1,1,2-trichloroethane (TCA) and benzene, with benzene 5.501 mg/L, CT 9.010 mg/L and TCA 1.200 mg/L. Other contaminants, predominantly comprising xylene and ethylbenzene, were at moderately low concentration and were omitted in the microcosm study. Consequently, the limited availability of electron acceptors, the negative ORP, and the high contaminant concentrations jointly indicate that the environment is favorable to anaerobic microbial degradation of contaminants [19].

### 3.2. CT Degradation Pathway

**Figure 2:**
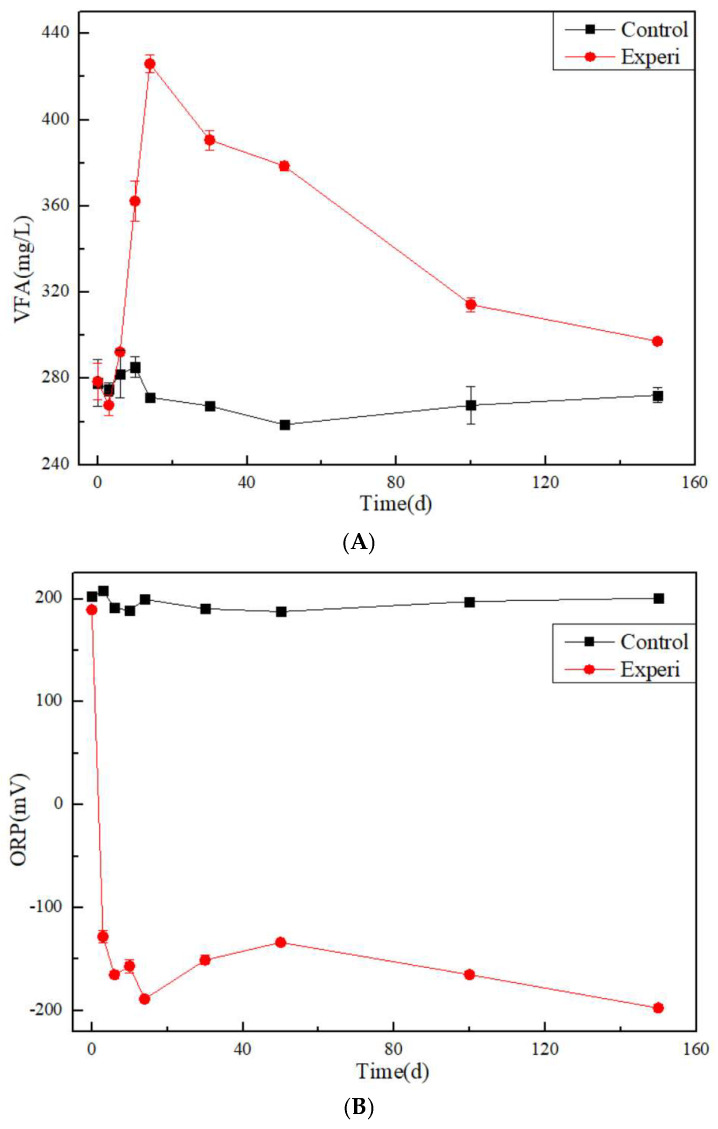

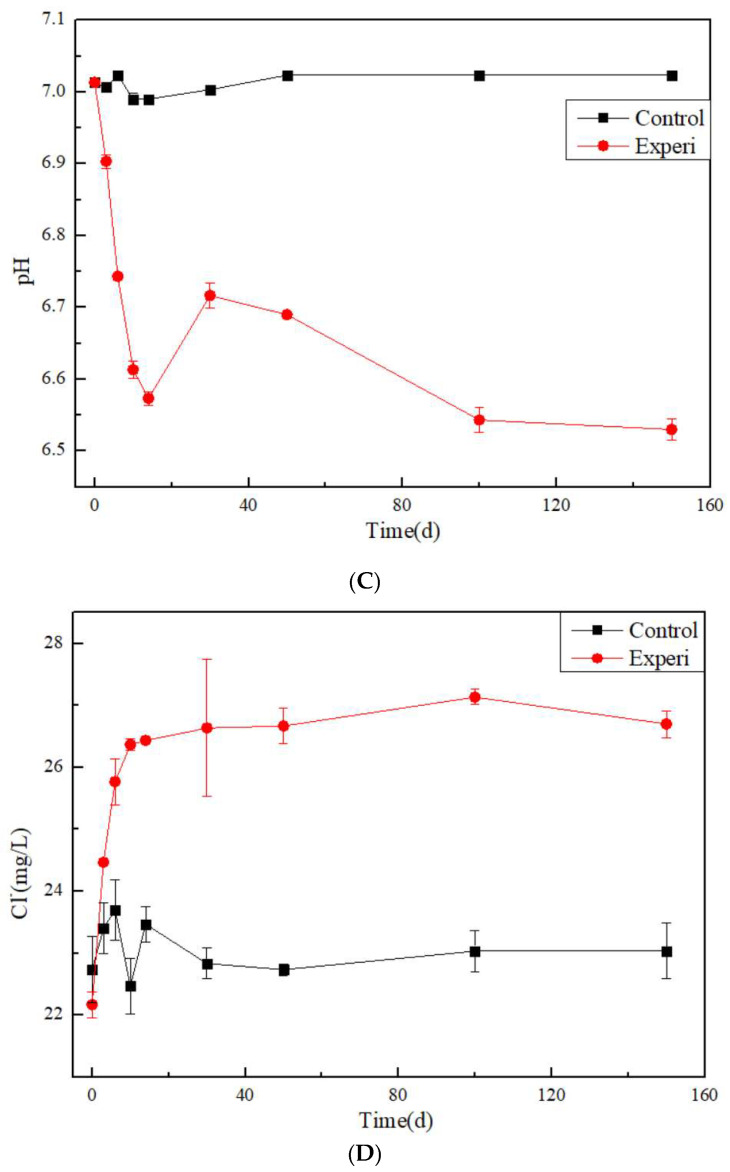
The changes in (**A**) VFAs, (**B**) ORP, (**C**) pH and (**D**) Cl^−^ in the Experi group and the control group.

The concentration of volatile fatty acid (VFA) in the control group remained basically unchanged at 280 mg/L in Figure 2. However, in the Experi group, the concentration of VFA rapidly increased within the first 10 days, reaching over 420 mg/L, and then gradually showed a downward trend. The reason for the increase in VFA is that yeast extract powder produces a large number of organic acids such as acetic acid and propionic acid during anaerobic fermentation.

Meanwhile, the ORP in the control group remained at around 200 mV. However, in the Experi group, the ORP rapidly decreased within the first 10 days, reaching −200 mV, and then gradually showed a slight fluctuation trend. The reason for the decrease in ORP value is that yeast extract consumes high-oxidation substances such as dissolved oxygen and nitrate during anaerobic fermentation, reducing to an absolute anaerobic state.

The pH in the control group remained at around 7. However, in the Experi group, the concentration of pH decreased within the first 10 days, reaching 6.55, and then gradually showed a slight fluctuation trend. The reason for the decrease in pH value is that yeast extract produces a large amount of organic matter acids such as acetic acid and propionic acid during anaerobic fermentation, which leads to a decrease in pH. This is also consistent with the increase in VFA data.

**Figure 3:**
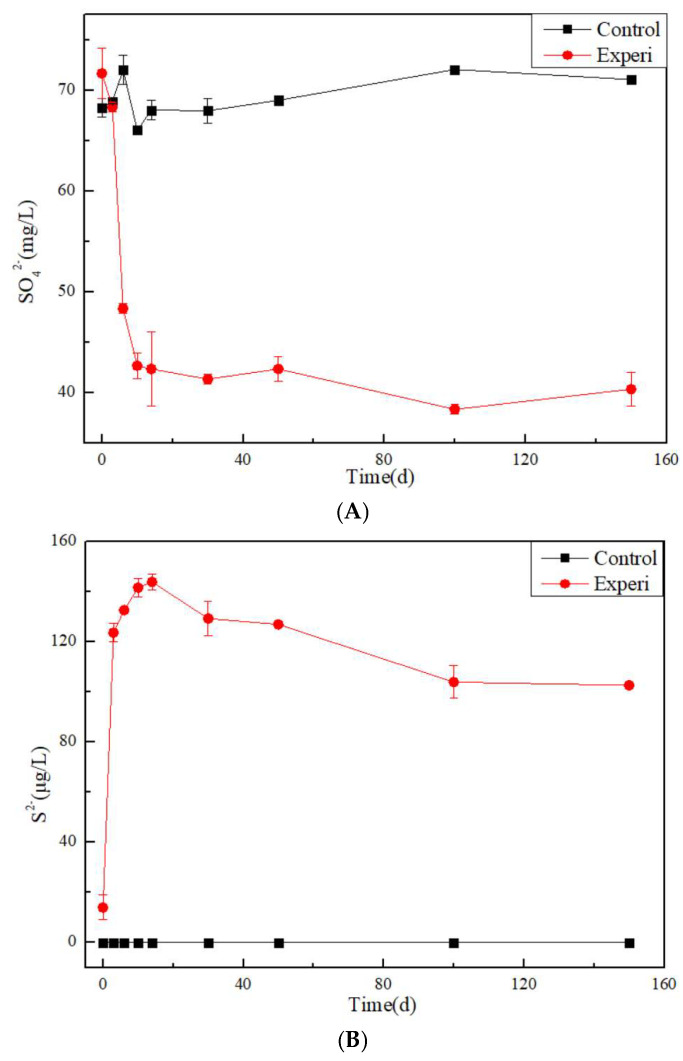
The changes in (**A**) SO_4_^2−^ and (**B**) S^2−^ in the Experi group and the control group.

The concentration of sulfate ions in the control group remained basically unchanged at 68 mg/L in Figure 3. However, in the Experi group, the concentration of sulfate ions rapidly decreased within the first 10 days, reaching 40 mg/L, and then gradually showed a stable trend. Meanwhile, the concentration of S^2−^ in the control group was maintained at 0 mg/L. However, in the Experi group, the concentration of S^2−^ rapidly increased within the first 10 days, reaching 140 µg/L, and then gradually showed a slight downward trend. This is due to the reduction of sulfate ions, which produce S^2−^.

**Figure 4:**
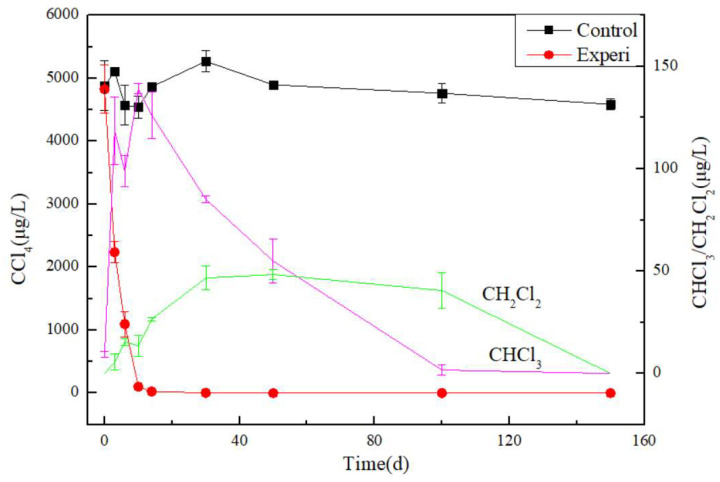
The concentrations of CT and its intermediate degradation products, CF and DCM.

The concentration of CT in the control Experi group remained basically unchanged in Figure 4. No CF or DCM was detected for the control groups. In comparison, the decreasing CT concentration value indicated that CT was biodegraded. The concentration of CT has been completely degraded by 40 days and cannot be detected anymore. In addition, the concentrations of CF and DCM contaminants increase first and then decrease. It is supposed that CF and DCM are speculated to be intermediate products in the degradation process of CT. It is reported that at least three competing pathways of CT degradation were identified, which have been well established in laboratory experiments and field pilot-scale testing. They are the abiotic hydrolysis, reductive dehalogenation and reductive hydrolysis pathways [13]. The degradation pathway in this paper is in accordance with the reductive dehalogenation pathway.

Meanwhile, the concentration of chloride ions in the aqueous solution remained constant in the control group in Figure 2. In the Experi group, its concentration increased rapidly in the early stage and gradually stabilized in the later stage. These data are also similar to the trend of contaminant concentration changes.

### 3.3. Microbial Community Composition

By aligning the 16S rRNA gene sequences to the SILVA 128/16 S database, the main genera were observed. The most abundant genera at the initial sample were *Pseudomonas*, *Nitrosomonas*, *Nitrospira*, *Methylobacter* and *Chlamydia*. However, cultures such as Desulfitobacterium, *Dehalobacter restrictus*, *Desulfuromonas*, *Dehalospirillum multivorans* and *Dehalococcoides*, that are known to be capable of dechlorinating PCE and TCE to cis-DCE so far, were not detected.

**Figure 5:**
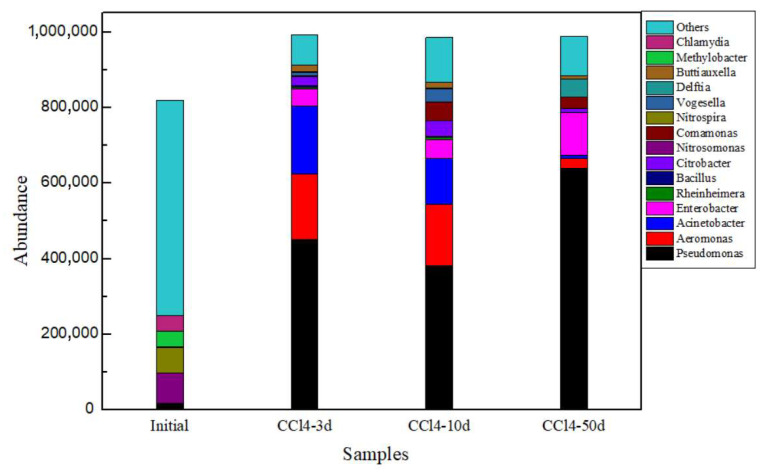
Changes in microbial community composition of samples in the Experi group.

As the reaction progressed, especially at 3 and 10 days, the microbial communities of the Experi samples were very similar in Figure 5. *Pseudomonas*, *Aeromonas*, *Acinetobacter* and *Enterobacter* dominated. At 50 days, there were slight differences in the microbial community compared to the 3- and 10-day samples, with an increase in the microbial communities of *Pseudomonas* and *Enterobacter*, and a decrease in microbial communities of *Aeromonas* and *Acinetobacter*.

According to Table 3, the Goods coverage indices of different samples were larger than 99%, which signified that the sequencing depth was sufficient. Groundwater samples from the initial sample exhibited the highest diversity with their Shannon and Simpson index values markedly higher than those from the other samples. Consequently, addition of YE and CT contaminant to the groundwater reduced the diversity and abundance of microbial community in the groundwater samples. These observations indicated that under the combined influence of the organic material and the presence of contaminants, the microbial community structure was dominated by anaerobic microorganisms capable of degrading CT compounds. CT degrading bacteria were stimulated as evidenced by the accelerated removal rate of the contaminant according to the degradation data.

**Figure 6:**
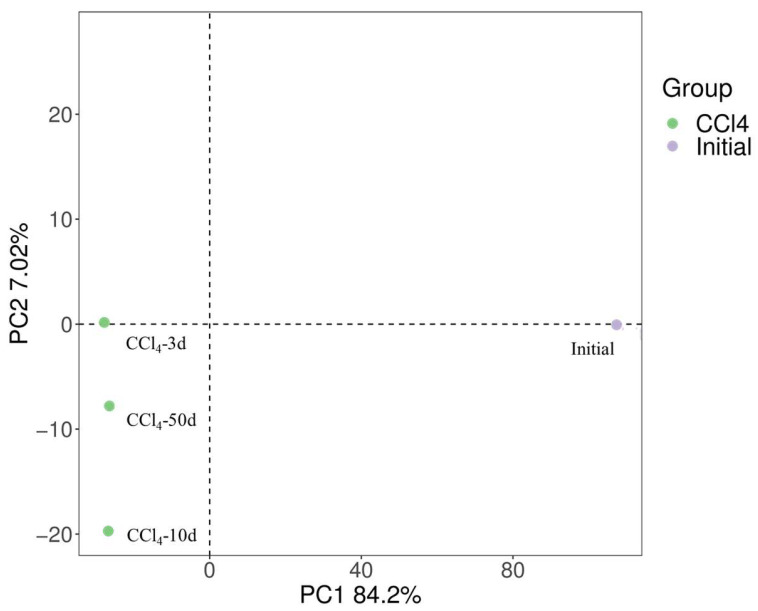
Two-dimensional PCoA of the samples in the Experi group.

To demonstrate the association between microbial structures in various samples, two-dimensional PCoA plots are displayed in Figure 6. It is well understood that the distances between samples are crucial for analysis within a PCoA plot [20]. Typically, samples that cluster closely together indicate a higher degree of similarity in their microbial community composition, while those that are more widely spaced suggest greater disparities in community structure. The CT sample at te 3, 10 and 50 days clustered closely together, indicating a high level of similarity in the microbial community structures. In contrast, the initial samples were notably distant from other samples on the PCoA plot, underlining a considerable variance in the respective microbial communities.

### 3.4. Functional Genes and Related Microorganisms

Researchers have documented hydrogen as an electron donor in anaerobic dechlorination [21,22,23,24]. Laboratory cultures applied to investigate anaerobic reductive dechlorination are usually mixed microbial consortium, with two different strains of bacteria as a minimum: one strain ferments the carbon-based substrate to yield hydrogen, and another strain consumes the hydrogen as an electron donor for anaerobic dechlorination. As hydrogen is generated by fermentative bacteria, it is quickly consumed by other bacteria, including iron-reducers, sulfate-reducers, methanogens, and dechlorinating microorganisms.

#### 3.4.1. Hydrogenase Genes

**Figure 7:**
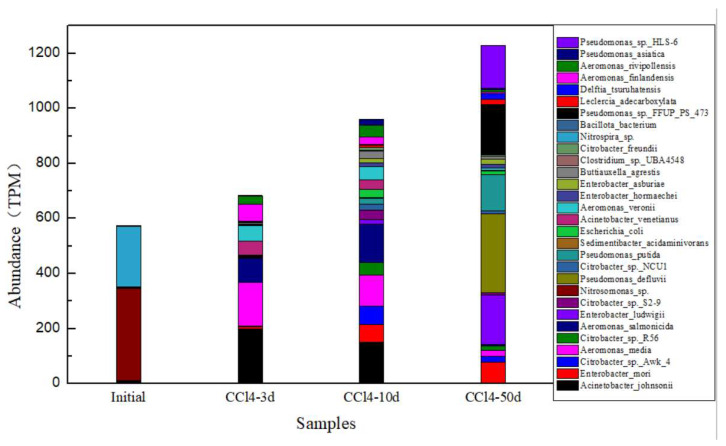
Identified hydrogenase gene bacteria and their abundances according to the KEGG database.

According to KEGG enzyme annotation results, hydrogenase genes may take responsibility for hydrogen generation. Annotating these hydrogenase genes to the Nr database, *Acinetobacter_johnsonii*, *Aeromonas_media*, *Enterobacter_mori*, *Citrobacter_sp._Awk_4, Aeromonas_salmonicida*, *Acinetobacter_venetianus*, *Aeromonas_veronii* and *Aeromonas_finlandensis* were the most abundant bacteria in Figure 7. During the range of 3 to 10 days of contaminant degradation, the most predominant species were *Acinetobacter_johnsonii* and *Aeromonas_media*. Many investigations have proposed that *Acinetobacter_johnsonii* could produce hydrogen [25,26], and it was often believed to be a collaborator for dehalogenation. In other studies, the production of H_2_ can be achieved through dark fermentation using the Acinetobacter junii-AH4 strain by optimizing different pH values and using different industrial wastewater as substrates. This indicates that Acinetobacter strains have potential in H_2_ production [27].

Hydrogenase gene abundances increased progressively with time. The abundance of *Acinetobacter_johnsonii* increased considerably from the 3-day sample and occupied a significant percentage of the total abundance all through the incubation.

At 50 days, the species *Enterobacter_ludwigii* and *Pseudomonas_sp._HLS-6* dominated. Researchers isolated the *Enterobacter ludwigii* strain from rice bran and characterized its hydrogen-producing efficiency [28]. This indicates that the *Enterobacter ludwigii* strain is a promising H_2_ production strain that can effectively utilize agricultural waste as a renewable biomass resource.

#### 3.4.2. Reductive Dehalogenase Genes

**Figure 8:**
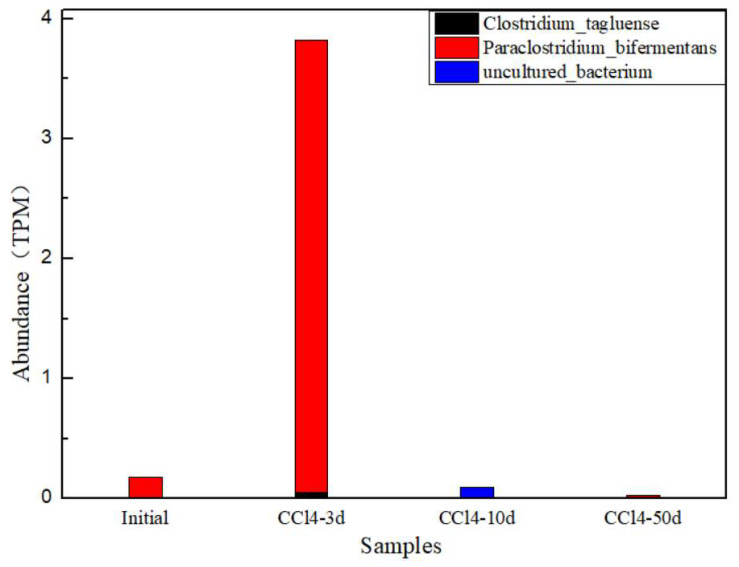
Identified reductive dehalogenase gene bacteria and their abundances according to the KEGG database.

Only three reductive dehalogenase species could align to PF13486 in the PFAM database. These species are annotated as *Clostridia tagluense*, *Paraclostridium_bifermentans* and uncultured_bacterium according to the Nr database in Figure 8.

This study has shown that the bacterium of the order Clostridiales, which is a planktonic microorganism, performs the reductive dechlorination of chloroform (CF) to DCM [3]. Further, the *Clostridium* strain can reduce CT to CF and further convert CF to DCM. These processes may occur under anaerobic conditions and may interact with the metabolic activities of other microorganisms [29]. Under anaerobic conditions, some Clostridium strains can form a co-metabolic relationship with dechlorinating bacteria (such as desulfitobacterium) to promote the reductive dechlorination of chlorinated organic contaminants.

*Paraclostridium* is a kind of strictly anaerobic bacteria, and their mechanism of action in dechlorination has not been clearly described in the search results. However, it can be speculated that they may participate in dechlorination through the sulfate reduction process. For example, some studies have shown that sulfate-reducing bacteria can reduce sulfate to hydrogen sulfide under anaerobic conditions and promote the dechlorination of organic contaminants. In this process, SRB may promote dechlorination by providing electron donors or directly participating in the reduction reaction of chlorinated compounds.

In the initial sample, the *Paraclostridium_bifermentans* exist in minimal amounts. At 3 days of incubation, the *Paraclostridium_bifermentans* began to increase, and the amount reached 3.8TPM. This verified that under the dual stimulation of chlorinated hydrocarbon contaminants and anaerobic environments, the microbial population capable of degrading chlorinated hydrocarbon contaminants has increased. It also aligned with the contaminant concentration change. Most of the contaminant was removed during the first 10 days.

### 3.5. Deduced Mechanisms of CT Biodegradation

It is known that the decomposition process of organic compounds is strongly related to their chemical structure and depends on the reaction conditions. The degradation pathway of CT can be precisely determined by the transformation of intermediates species and the concentration [14]. Not only CF was observed, but DCM also existed at trace levels in this study. The formation of CF and DCM suggested that sequential dechlorination is the main degradation pathway in the CT anaerobic conversion. This study has indicated the generation of green rusts precipitates as a byproduct of the biogenic activity of A. suillum and the role of these Fe-minerals as natural reducing agents of CT to form CF, DCM and CM (and possibly CH4) [3].

According to the intermediate species, the anaerobic degradation paths of CT may possibly be summarized as two key stages. Initially, the dissociative electron transfer reaction in the CT took place to yield trichloromethyl free radicals. Then, via reduction and protonation of ⋅CCl_3_, the CF product was produced. Essentially, the electron cloud density distribution of H atoms was weaker than that of Cl atoms, resulting in less repulsion between the H atom and other atoms, which promoted the covalent bond formation between the H atom and C atom. In addition, the reductive dechlorination of chlorinated hydrocarbons is an integral form of chemical oxidation and chemical reduction reactions under the action of microorganisms. Therefore, the reaction is shown in Equations (1) and (2):

(1) CT + e^−^ → Cl^−^ + CCl_3_

(2) ⋅CCl_3_ + [H] → HCCl_3_

Although DCM is only a product at a modest concentration in this study, its presence offered obvious evidence for the sequential dechlorination in the microcosm. Therefore, the further conversion reaction was shown in Equation (3):

(3) HCCl_3_ +e^−^ → Cl^−^ + HCCl_2_

(4) HCCl_2_ + [H] → H_2_CCl_2_

Through the steps above, CT could be decomposed by the dichlorination bacteria. Moreover, according to the concentration change trend chart of CT, CF and DCM in Figure 4, it can be inferred that the biodegradation rate of CT is the fastest, followed by CF, and the slowest is DCM, with the lowest concentration change, but showing a very slow degradation curve. Therefore, the slow biodegradation of CF and DCM needs special attention, especially when CT contaminants are degraded by on-site biological stimulation.

In this study, YE acted as the carbon source and operated as the energy source. During metabolism, the high-molecular-weight constituents of YE were transformed into low-molecular-weight constituents, such as VFAs, acetate and H_2_, offering substrates applied for CT biodegradation.

From a microbial point of view, the bacteria containing hydrogenase (EC. 1.12.99.6) genes responsible for hydrogen production, including *Acinetobacter_johnsonii*, *Aeromonas_media*, *Enterobacter_mori*, *Citrobacter_sp._Awk_4*, *Aeromonas_salmonicida*, *Acinetobacter_venetianus*, *Aeromonas_veronii* and *Aeromonas_finlandensis*, are abundant in the microbial culture. The functional genes were more abundant in the later phases.

The bacteria containing reductive dehalogenase genes responsible for dehalogenase production, including *Clostridia tagluense*, *Paraclostridium_bifermentans* and uncultured_bacterium, is relatively low. At 3 day of incubation, the *Paraclostridium_bifermentans* began to increase, whose amount reached 3.8TPM. the *Paraclostridium_bifermentans* might play the key role in the dehalogenase production, and also in the biodegradation of chlorinated hydrocarbons. It also verified that under the dual stimulation of chlorinated hydrocarbon contaminants and anaerobic environments, the microbial population capable of degrading chlorinated hydrocarbon contaminants has increased.

In conclusion, the CT biodegradation-related microbes are inferred as follows. The CT dehalogenation electron donor, H_2_, was generated by *Acinetobacter_johnsonii*, *Aeromonas_media*, *Enterobacter_mori*, *Citrobacter_sp._Awk_4*, *Aeromonas_salmonicida*, *Acinetobacter_venetianus*, *Aeromonas_veronii*, *Aeromonas_finlandensis* and other hydrogen producers through fermentation of YE. *Clostridia tagluense*, *Paraclostridium_bifermentans* and other suspected dehalogenators may synthesize Rdhs to sequentially reduce CT to CF to DCM. The mechanisms above are based on concepts of other CAH biodegradation processes, and H_2_ served as the electron donor. CT reduction and H_2_ oxidation are deemed half-reactions of the same biochemical process.

## 4. Conclusions

This study presents a comprehensive investigation into the bioremediation of CT-contaminated groundwater using ERD. The application of yeast extract as an additional electron donor facilitated the degradation of CT, achieving complete removal within a 40-day period in the experimental setup. The physical and chemical indexes such as VFAs, sulfate ions, oxidation–reduction potential and pH value reveal that strong reducing conditions are created and the chlorinated hydrocarbons’ reductive dechlorination can be promoted. The detection of CF and DCM suggests that sequential dechlorination is the main degradation pathway in the anaerobic transformation of CT. This study’s findings underscore the significance of specific microbial genera, such as *Acinetobacter*, *Aeromonas* and *Enterobacter*, which are implicated in hydrogen production, a key electron donor for the reductive dechlorination of CT. *Clostridia tagluense*, *Paraclostridium_bifermentans* and other suspected dehalogenators may synthesize Rdhs to catalytically reduce CT to CF to DCM.

This study’s results not only promote a better interpretation of the complex interactions within contaminated groundwater ecosystems but also offer practical implications for developing effective bioremediation approaches to address CT and other chlorinated hydrocarbon contaminants. Future research should focus on scaling-up these findings to field applications and exploring the long-term stability and efficiency of the observed microbial consortia in diverse environmental conditions.

## Figures and Tables

**Table 1:** Components of the microcosm samples.

Microcosm Sample	Components
Control	deionized water + contaminated groundwater containing CT (5 mg/L) + YE (1 g/L) + 10 mg/L HgCl_2_
Experi	deionized water + contaminated groundwater containing CT (5 mg/L) + YE (1 g/L)

**Table 2:** Physiochemical properties of the collected groundwaters used in the microcosm samples.

pH	ORP (mV)	DO (mg/L)	NO_3_^−^ (mg/L)	Fe^2+^ (mg/L)	Mn^2+^ (mg/L)	CT (mg/L)	TCA (mg/L)	Benzene (mg/L)
6.87	−38	0.55	0.882	5.40	3.55	9.010	1.200	5.501

**Table 3:** The richness and alpha diversity estimator of microbial community.

Sample	Chao1	Goods Coverage	Shannon	Simpson	Ace
Initial	25,267.41	0.9999	5.9333	0.9837	24,542.15
Experi-3d	12,244.47	0.9999	4.7413	0.9572	11,867.95
Experi-10d	13,410.79	0.9999	5.0467	0.9765	12,811.84
Experi-50d	10,510.39	0.9999	3.8062	0.9207	10,029.00

## Data Availability

The data presented in this study are available on request from the corresponding author.
